# Burnout in Spanish Security Forces during the COVID-19 Pandemic

**DOI:** 10.3390/ijerph17238790

**Published:** 2020-11-26

**Authors:** José Gómez-Galán, Cristina Lázaro-Pérez, Jose Ángel Martínez-López, María del Mar Fernández-Martínez

**Affiliations:** 1 Department of Education, University of Extremadura, Avda. de Elvas, s/n, 06006 Badajoz, Spain; 2 College of Education, Ana G. Méndez University, Cupey Campus, San Juan, PR 00926, USA; 3 Department of Sociology, University of Murcia, Avda. Teniente Flomesta, 5, 30003 Murcia, Spain; cristina.lazaro2@um.es; 4 Department of Social Work and Social Services, University of Murcia, Avda. Teniente Flomesta, 5, 30003 Murcia, Spain; jaml@um.es; 5 College of Education Sciences & College of Sociology, Social Work and Public Health, University of Huelva, Campus El Carmen, Avda. de las Fuerzas Armadas, s/n, 21007 Huelva, Spain; mar.fernandez@dstso.uhu.es

**Keywords:** burnout, COVID-19, police, armed forces, state security forces, anxiety, prevention

## Abstract

Since the beginning of the COVID-19 pandemic in Spain, members of the State Security Forces and the Armed Forces have been mobilized to guarantee the security and mobility of the population and to support health institutions by providing personnel for care, creating field hospitals, transferring the sick and the dead, etc. The objective of this study was to determine the levels of burnout in these professionals using the Maslach Burnout Inventory (MBI) scale, both in its different subscales and its total value. The study was developed using a quantitative methodology through a simple random sample (*n* = 2182). An ad hoc questionnaire was administered including variables related to: (a) socio-demographic issues, (b) subjective perceptions about their working conditions and the need for psychological and psychiatric treatment, and (c) the Death Anxiety Scale developed by Collett–Lester, and the MBI. The results show high levels of burnout (28.5%) in all its subscales: emotional exhaustion (53.8%), depersonalization (58.0%), and lack of personal development (46.3%). The logistic regression verifies a series of predictive variables that coincide in each of the subscales. These data indicate the need to implement prevention and treatment measures for workers so that their, stress, and anxiety to which they are subjected during their professional activity does not become a norm that can have negative repercussions for them, especially given the risk of new pandemic waves.

## 1. Introduction

In Spain, three entities integrate the set of corps and forces linked to different ministries that monitor the defense, protection, and security of the country and all its citizens: the Armed Forces (*Fuerzas Armadas*: FFAA), the State Security Forces and Corps (*Fuerzas y Cuerpos de Seguridad del Estado*: FFCCSE), and the Security Forces and Corps belonging to the Ministries of the Interior and Defense (*Fuerzas y Cuerpos de Seguridad pertenecientes a los Ministerios del Interior y de Defensa*: FFCCS). The Armed Forces (FFAA), “integrated into the Ministry of Defense, are [an] essential element of the national defense, and make up a unique entity that is conceived as an integrating set of the specific forms of action of each of its components: the Army, the Navy and the Air Force” [1]. They are organized in an organic structure designed to prepare the Force and other operative entities dedicated to the missions assigned to them. The mission of the State Security Forces and Corps (FFCCSE) is to protect the free exercise of rights and freedoms and to guarantee citizen security. They are composed of two corps part of the Ministry of the Interior: the National Police Force (*Policía Nacional*), which is an armed institute of a civilian nature, and the *Guardia Civil*, which is an armed institute of a military nature, which also performs missions of a military nature. In times of war and during a state of siege reports exclusively to the Ministry of Defense [2]. In times of war and during a state of siege, reports exclusively to the Ministry of Defense Finally, the Forces and Corps of Security (FFCCS), integrating the forces and corps for the security of the state, are dependent on the national government, the corps of the police of the autonomous communities, and the corps of police of the local corporations. These participate in the “maintenance of public security in the terms established in the Law regulating the Bases of Local Regime and in the framework of this Law” [2], which is exercised by the public administrations through the security forces and corps.

The labor performed by the state security forces, especially the militarized corps, influences the level of stress of its members, which harms their health [3]. Continuous and prolonged exposure to stress may result in the appearance of burnout [4]. Essential workers who perform their labor in challenging contexts, such as those previously mentioned, have the responsibility to make difficult decisions; they express concerns about their exposure and that of their families [5,6], which may have a potentially negative impact on their mental health in the short and long terms [7].

Maslach and Jackson [8] defined burnout as a syndrome characterized by emotional exhaustion, depersonalization, and low self-realization at work, which often occurs in people whose daily tasks are established in the service of people.

Identifying the most prominent stressors has been a difficult task for researchers, but some authors noted two types: organizational (produced by the police administration and management) and inherent (originating from frequent occupation, which harmful to their physical and psychological health, such as exposure to danger and brutality) [9,10,11]. Later, others differentiated the stressors into four groups based on the sources of risk in this profession [12]: management, the performance of tasks, the community, and the judicial system. These effects of the stress and stressors are not exclusive to the police profession but are faced by other related professions such as the armed forces and, in general, all the state security forces and corps [3,13,14,15,16,17]. This is also the case with death anxiety, which has been able to develop in overflowing situations very close to death and which could interfere with professional development since it is not possible to identify it at the time [18].

The health crisis caused by the emergence of the COVID-19 pandemic (Coronavirus Disease), an infectious disease caused by the novel coronavirus SARS-CoV-2 (2019-nCoV), has affected all sectors of the population in Spain, especially those who have worked on the front line such as health care professionals and organizations working for the safety and protection of citizens [5,18]. These professions, considered essential, have not been exempt from the stress caused by COVID-19 in society; they have had to perform their work anomalously due to the pandemic, experiencing alterations in their usual tasks. In these circumstances, the Armed Forces, the FFCCSE, and the FFCCS have had to work, facing a situation that could affect their mental health and they have been at greater risk of stress because of the long exposure to the virus [19,20,21] and the new policies adopted to improve coexistence and protection of citizens when they needed them most. In this situation, there are two vital aspects to consider: if, in this pandemic context, the stress factors related to work changed, and if the level of stress of the security forces has increased [19].Under these circumstances, while the population was kept in confinement after the declaration of a state of alarm by the Spanish government, these workers had to continue working, while new stressors emerged: new protocols of action, changes in shifts, lack of personal protective equipment (PPE, including lack of hydroalcoholic gels and gloves), infected colleagues, lack of PCR (polymerase chain reaction) tests, and an increasing number of deaths every day. This was compounded by the Ministry of Health reporting a low probability of exposure to SARS-CoV-2 [22] or the avoidance by the Ministry of the Interior to commit to the measures proposed by the Unified Association of Civil Guards [23] or to recognize them as high-risk personnel. The forces experienced all this while still being required by the government [24]. The figures at the beginning of the pandemic showed that over 2300 national police and civil guards (1583 national police and 734 civil guards) were quarantining in their homes for symptoms compatible with COVID-19, 94 had confirmed positive for COVID-19 (60 in the police and 34 in the civil guard) as of 19 March 2020, and 19 died in the FFCCS according to official government figures as of 29 April 2020 [25]. However, the figures reported by the AUGC [23] on 16 April 2020 provided infection numbers of 1483 civil guards, 751 national police, and 868 local police. In terms of the number of isolated individuals, on that date, there were 2384 civil guards, 2360 national police, and 1671 local police. These figures continued to increase.

The stress level experienced by protection and advocacy professionals may have altered during the COVID-19 pandemic when intervening with persons with mental health problems exacerbated by fear of contagion, resource scarcity, economic uncertainty, and social alienation [19].

Since the end of the confinement in Spain on 21 June 2020, the relaxation of the citizenry, including non-compliance with health rules and restrictions, has increased the number of police interventions and the mass contact with possible transmitters [18]. Likewise, the second wave of the pandemic brought an increase in active cases and hospital admissions exceeding the cases located during the first wave in a one-month period.

All situations of psychosocial risk [26,27,28,29,30], which has coincided in Spain with numerous environmental disasters such as fires and floods, could once again generate situations of maximum stress. In a very short time period, both the civilian population and the professionals who compose the FFCCS have had to face a heavy workload, with the constant threat of possible contagion by the SARS-CoV-2, without rest since March 2020.

## 2. Materials and Methods

### 2.1. Objectives

The unprecedented situation faced by the members of the State Security Forces and Corps and the armed forces makes it necessary to consider these professionals, showing how the current health crisis has affected them from the dimension of burnout [8]. In this research, the general objective (GO) was to take a deep approach to the phenomenon of burnout in these professionals, contextualized in the temporary period during the first wave of COVID-19 in Spain. This GO is broken down into the following specific objectives (SOs): to quantify the general level of burnout and the set of subscales of the Maslach Burnout Inventory (MBI) scale (SO1), and to determine the predictive variables that influence these levels of burnout in these professionals (SO2).

### 2.2. Research Instrument

The research instrument was a questionnaire composed of several blocks to measure burnout in the State Security Forces and Corps and the Armed Forces based on the Maslach Burnout Inventory (MBI) developed by Maslach and Jackson [8] and validated in Spanish by García et al. [31]. This scale allows us to determine the general level of burnout, and to identify the existence of burnout through its three dimensions made up of subscales: Emotional Exhaustion (EE), Depersonalization (DP), and Personal Accomplishment (PA). The MBI is composed of 22 items with 7 response options through a Likert scale whose values range from 0 to 6. The AE scale is composed of 7 items, the DP of 5, and PA of 8. EE is considered low when its values are between 0 and 18, medium when they are between 19 and 26, and high when between 27 and 54. Regarding PA, low scores range between 0 and 5, average between 6 and 9, and high between 10 and 30. Finally, with regards to PA, a low score registers values between 0 and 33, medium between 34 and 39, and high between 40 and 56. For the overall burnout assessment, there must be high levels of EE and DP and a low level of PA.

Besides this burnout block, the questionnaire included: (a) questions regarding socio-demographic issues, (b) subjective perceptions about their working conditions and the need for psychological and psychiatric treatment, and (c) the Death Anxiety Scale developed by Collett-Lester [32] composed of 4 subscales on “Fear of one’s Death”, “Fear of the Process of Dying One’s Own”, “Fear of the Death of Others” and “Fear of the Process of Dying Others” with 7 items each, and a total of 28 items. The answers are of the Likert type from 1 (nothing) to 5 (a lot). This scale was included in the research as the increase in mortality during the first wave of the pandemic was very high in Spain and could provide a predictive variable of the burnout of these professionals.

### 2.3. Participants

There were 2182 participants in the research; their main characteristics are shown in Table 1. The majority of the sample was composed of men, representing 87.3% of the total, and women representing just 12.7%. In terms of age, the majority group was between 31 and 40 or 41 and 50 years old, at 36.6% each, totaling 73.2%. Regarding the workplace, 46.1% were National Police (*Policía Nacional*), 36.8% Civil Guards (*Guardia Civil*), 17.2% military, 3.9% local police, and 0.5% were other (autonomous and local police forces in the Basque Country and Catalonia or security agents).

In this descriptive study, the approval of the ethics committees of the Spanish universities of the researchers was not necessary (it is only required for experimental research). In our case, we subscribed to all the Codes of Good Practices of the Ethics Committees regarding research on human beings. The study was registered (code No. REPRIN-PEM-15B) and signed by the research team that performed the entire research process. The guidelines set by the Declaration of Helsinki were followed; all participants (*n* = 2182) provided their informed consent.

### 2.4. Variables

The dependent variable in this research was the burnout levels, both with the general index and of each one of its subscales: EE, DP, and PA.

For the independent variables, the following were used: (a) sex; (b) age; (c) professional body; (d) subjective perception of needing psychological/psychiatric treatment because of COVID-19; (e) assessment of whether work centers should offer psychological/psychiatric support as a consequence of COVID-19; (f) perception of need for psychological/psychiatric support; (g) assessment of whether the lack of PPE increased stress or anxiety level; (h) level of death anxiety (from the Collett–Lester scale); (i) whether they worked directly in COVID-19 tasks during the first wave of the pandemic in Spain; (j) whether they felt professional recognition during the first wave of the pandemic in Spain.

### 2.5. Procedure

The field research was conducted between 7 August and 7 September 2020. We accessed the participants through the collaboration of several professional associations and Spanish trade unions who were asked to participate in the questionnaire’s dissemination. Initially, their collaboration was requested to participate in the research. Subsequently, these entities sent a link to access the questionnaire through a platform designed ad hoc by the University of Murcia for the preparation, administration, completion, and exploitation of statistical studies developed through surveys.

The following phases were followed in the process of data analysis: Initially, a frequency analysis was performed to determine the general level of burnout and each one of its subscales. Later, to obtain information about the variables linked to this phenomenon, a cross-table analysis was performing considering a chi-square *p* ≤ 0.05. Finally, to determine the variables predictive of the phenomenon, binary logistic regressions were performed, taking the existence of the general burnout level, and the medium-high level of each one of the EE, DP, and PA subscales as reference variables.

The variables used in the binary logistic regression are shown in Table 2.

## 3. Results

First, the burnout levels of the Security Forces and Corps are high, both in terms of the subscales and the general burnout level. In the first case, the Emotional Exhaustion subscale showed a high level in 53.8% of participants (70.1% with medium-high level). The Depersonalization subscale showed levels even higher than EE, at 58.0% (reaching a high percentage if we take the medium-high level of 85.5% as a reference). The Personal Accomplishment subscale was low (taken as a reference), reaching 46.3% (72.9% if considering the low-medium level). Finally, 28.5% of the total were found to be suffering from burnout, showing high levels of emotional exhaustion and depersonalization, and low levels of personal accomplishment (Table 3).

For the rest of the variables, 69.1% of the participants reported high levels of death anxiety. A total of 26.2% reported the subjective perception of needing psychological treatment. These data are similar to the percentage of people with burnout levels on the general scale (28.5%). A high percentage of respondents (88.2%) considered it necessary to provide psychological or psychiatric treatment from work centers because of COVID-19, and 52.5% reported they may need new psychological treatment in the future due to COVID-19, which is twice as many as those who state that they need it at present (26.2%).

A total of 87.2% considered that the lack of PPE during the most virulent weeks of the pandemic in the first wave in Spain increased their level of stress and/or anxiety. Almost 9 out of 10 people saw their level of stress and/or anxiety increase at work as a result of not having the basic supplies for their personal protection. Of the participants, 73.9% worked directly in a COVID-19 environment and 89.6% reported that they have not felt recognized in their professional activity by the institution in which they work. These percentages within this section of subjective assessment are high and demonstrate the position and disposition that the members of these corps may show in their professional activity, and how the context and working conditions may affect their psychological and social status.

Concerning the binary logistic regression applied in all the MBI subscales and to the total burnout index, adequately adjusted, robust, and reliable models were obtained. Each of the models is developed below.

The binary logistic regression of the Emotional Exhaustion subscale was found to be a statistically significant model (χ^2^ = 301.911, *p* < 0.000). The model explained 18.9% (Nagelkerke’s *R*^2^) of the variance of moderate-high consumption and correctly classified 74.2% of the cases. The Hosmer–Lemeshow test showed that there were no significant differences between the observed and predicted results in the model (*p* = 0.800).

The variables included in the equation were: age, professional body, subjective perception of needing psychological/psychiatric treatment as a consequence of COVID-19 (NPPS), perception of whether they believe they may need psychological/psychiatric support (PPSN), assessment of whether the lack of PPE increased their level of stress or anxiety (PPE), high level of anxiety in the face of death (DA), and whether they felt professional recognition during the first wave of the pandemic in Spain (FWR).

Regarding age, the interval between 41 and 50 years had an odds ratio (OR) of 1.422 (95% CI, 1062–1904, *p* = 0.018). Those in the middle age group reported up to 1.4 times more emotional exhaustion than younger people (up to 30 years). As this was the only value within the age category that showed representativeness, we found this age group was at the highest risk of suffering emotional exhaustion. All professional categories showed high levels concerning the reference category (others). The results were high in all cases. For the military, OR = 6678, 95% CI; 1175–37,941, and *p* = 0.032. For the National Police, OR = 9342, 95% CI: 1660–52,589, and *p* = 0.011. For the *Guardia Civil*, OR = 7670, 95% CI: 1362–43,216, and *p* = 0.021. Finally, the local police recorded an OR of 8457, a 95% CI of 1400–51,092, and *p* = 0.018. Therefore, these professional bodies reported high levels compared to the reference level (others), with the national and local police standing out as suffering from emotional exhaustion at 9.3 and 8.4 times higher levels, respectively. The *Guardia Civil* and the military registered 7.6 and 6.6 times higher possibilities of suffering emotional exhaustion than the reference value, respectively.

Concerning subjective issues, NPPS presented an OR of 1.642, 95% CI: 1207–2234, and *p* = 0.002. Needing psychological/psychiatric support increased the risk of suffering from emotional exhaustion by 1.6 times. The PPSN registered an OR of 2.002 (95% CI: 1.570–2.552, *p* = 0.000); so, people who reported that they may need future psychological/psychiatric treatment were up to two times more likely to suffer emotional exhaustion than those who did not report this need. PPE had an OR of 1.585, 95% CI: 1.187–2.116, and *p* = 0,002. Therefore, people who reported that the lack of PPE has influenced their health were up to 1.5 times more predisposed to suffering EE. DA registered an OR of 2.759 (95% CI: 2.238–3.402, *p* = 0.000). This is one of the highest values within this subscale, along with the professional categories, and showed how DA increases the possibility of suffering EE by up to 2.7 times. FWR had an OR of 0.678, 95% CI: 0.494–0.932, and *p* = 0.017. Because the B was negative (−0.388), people who did not feel recognized for their work in the first wave of the pandemic were more likely to suffer.

The binary logistic regression of the Depersonalization subscale showed a statistically significant model (χ^2^ = 127,948, *p* < 0.000). The model explained 10.1% (Nagelkerke’s *R*^2^) of the moderate-high consumption variance and correctly classified 83.0% of the cases. The Hosmer–Lemeshow test showed that there were no significant differences between the observed and predicted results in the model (*p* = 0.411).

The variables included in the equation were: sex, perception of whether the respondent believed they may need psychological/psychiatric support (PPSN), high level of death anxiety (DA), and whether they had felt professional recognition during the first wave of the pandemic in Spain (FWR).

Regarding sex, men had an OR of 1.744 95% CI: 1269–2398, and *p* = 0.001. Thus, men were almost twice as likely to suffer depersonalization as women. However, these data should be treated with caution since the sample is mainly composed of men. PPSN registered an OR of 1380, 95% CI: 1083–1758, and *p* = 0.009. Therefore, a connection exists between depersonalization and the perception of needing psychological/psychiatric treatment; people with this perception were almost 1.4 times more likely to report high values in this subscale. DA had an OR of 2.220, 95% CI: 1.740–2.832, and *p* = 0.000. Thus, the risk of suffering depersonalization was 2.2 times higher among people who had high values of AM. Finally, FWR recorded an OR of 0.653, 95% CI: 0.463–0.922, and *p* = 0.015. As with the Emotional Exhaustion subscale, its B was negative (–0.426), showing that people who did not felt recognized during the pandemic were more likely to suffer depersonalization.

For the Personal Accomplishment subscale, the binary logistic regression presented a statistically significant model (χ^2^ = 160,786, *p* < 0.000). The model explained 9.7% (Nagelkerke’s *R*^2^) of the variance of moderately high consumption and correctly classified 62.4% of the cases. The Hosmer–Lemeshow test showed that there were no significant differences between the observed and predicted results in the model (*p* = 0.525).

The variables included in the PA equation, as with the Personal Accomplishment subscale, were: sex, age, subjective perception of needing psychological/psychiatric treatment because of COVID-19 (NPPS), subjective perception of whether psychological or psychiatric support should be offered from workplaces (PPSS), perception about whether they believe they may need psychological/psychiatric support (PPSN), whether the lack of PPE increased their level of stress or anxiety (PPE), high level of anxiety in the face of death (DA), and whether they have felt professional recognition during the first wave of the pandemic in Spain (FWR).

With respect to sex, men had an OR of 0.727, 95% CI: 0.553–0.957, and *p =* 0.023. Their negative B (–0.318) indicated that being a woman reduces the possibilities of personal fulfillment, although the values were small and the female population is significantly less. In relation to age, people between 31 and 41 years old had an OR of 0.679, 95% CI: 0.523–0.881, and *p* = 0.004. As with the sex variable, B was negative (–0.519), so people in this age range were more likely not to suffer from a lack of professional fulfillment compared to the reference value (age up to 30 years).

Regarding the subjective variables, the NPPS recorded an OR of 0.595, 95% CI: 0.470–0.753, and *p* = 0.000. As with the previous variables, its B was negative (−0.519), showing that people who do not need psychological or psychiatric support had a greater chance of reporting positive values on this subscale. The PPSS had an OR of 1.593, 95% CI: 1.190–2.132, and *p* = 0.002. Thus, people who reported the need for psychological/psychiatric treatment in the workplace showed higher levels, up to 1.5 times more in this subscale, than those who thought this type of treatment should not be provided. PPSN registered an OR of 0.718, 95% CI 0.577–0.894, and *p* = 0.003. As in previous cases, its B was negative (–0.331), indicating that people who claimed not needing psychological/psychiatric treatment were more likely to be represented in this subscale. PPE recorded an OR of 0.611, 95% CI: 0.454–0.823, and *p* = 0.001. Its B was also negative (–0.492), so people who did not consider the absence of PID as increasing their stress/anxiety levels were more likely to be represented in this subscale. DA had an OR of 0.604, 95% CI: 0.495–0.738, and *p* = 0.000. Their B, as in most cases in this subscale, had negative values (–0.504), showing that the professional performance of those who did not report suffering from DA may have been more affected. Finally, FWR showed an OR of 1.304, 95% CI: 1.046–1.624, and *p* = 0.018. Thus, the professional performance of those who reported feeling recognized for their work during the first wave of the pandemic may have been affected up to 1.3 times more than the rest.

Finally, approximating the Total MBI, we observed that the binary logistic regression presented a statistically significant model (χ^2^ = 160,829, *p* < 0.000). The model explained 10.4% (Nagelkerke’s *R*^2^) of the moderate-high variance in consumption and correctly classified 71.0% of the cases. The Hosmer–Lemeshow test showed that there were no significant differences between the observed and predicted results in the model (*p* = 0.656).

The variables included in the equation were: age, NPPS, PPSS, PPSN, and DA.

Those aged 31–40 years recorded an OR of 1606, 95% CI: 1194–2159, and *p* = 0.002. NAPP presented an OR of 1888, 95% CI: 1479–2409, and *p* = 0.000. Thus, NPPS increases the chances of suffering burnout by people who consider that they need psychological/psychiatric support by almost two times PPSS showed an OR of 0.678, 95% CI: 0.489–0.940, and *p* = 0.020, although B was negative (–0.389). This result showed that people who consider that psychological/psychiatric support should not be provided in their workplaces have a greater predisposition to suffer burnout. Subsequently, PPSS recorded an OR of 1630, 95% CI: 1277–2082, and *p* = 0.000. Those who reported that they may need psychological or psychiatric treatment were at 1.6 times greater risk of burnout. Finally, DA presented an OR of 2.236, 95% CI: 1.762–2.837, and *p* = 0.000. This result indicated that people who have a high level of DA experience up to 2.2 times more burnout. For an overview, see Table 4.

## 4. Discussion

One consequence of the COVID-19 health crisis has been the proliferation of studies related to clinical research to generate methods for the disease’s treatment including the contextual and psychological effects derived from the pandemic. In this sense, we must determine the consequences of the pandemic on other levels [33,34,35,36], including the emotional and psychological levels.

Our findings showed the concern of security professionals regarding their mental health status. Of our respondents, 26.2% felt that they needed psychological treatment in the first wave of the pandemic, and 52.5% felt that they might need psychological treatment again.

We found that 28.5% of those surveyed reported a high level of burnout and high levels on the subscales of Emotional Exhaustion (53.8%) and Depersonalization (58.0%), and low levels of lack of personal fulfillment (46.3%). Similar results were found in a study in Italy [37], where there was no analysis of the protective elements that could protect these workers from the effect of burnout or of the weakening of the reasons for living and dehumanization.

Despite the measures implemented to contain mobility and improve hygiene, these groups have had to face the situation with caution and adopt new protocols [38] as in other countries [39]. One of these newly implemented safety standards was the use of PPE. Our study showed that 87.2% of the participants reported their level of stress and/or anxiety increased in their work because of not having the basic supplies for their personal protection. This is one of the reasons why the levels of death anxiety and burnout were elevated, as also reported in previous studies [5,21].

Considering the parameters of the Maslach Burnout Scale [8], we found that 28.5% of the people interviewed presented high levels of burnout with high values on the subscales of Emotional Exhaustion and Depersonalization, and low values in Personal Achievement. This suggests that although there were high scores in the previous subscales, the sense of achievement and self-efficacy were high, indicating great professional involvement. This result agrees with those of other European studies [37,40,41] considering the existence of burnout, although the percentage we found is much higher, suggesting that the context of the COVID-19 pandemic accentuates the number of people affected. Our findings contradict those of other studies such as one conducted in another context and in other countries, which find low job satisfaction in a group of police officers [42].

Although for decades multiple studies have determined the presence of stress and burnout in the security forces, in all professional areas [43,44,45,46,47,48,49,50], and sometimes with very critical consequences [51], the pandemic caused by SARS-CoV-2 has created an exceptional and extreme scenario that could significantly increase these problems [52]. Practically since the Spanish flu of 1918–1919, the world has not experienced a similar impact caused by a pathogen [53,54]. Studies are needed to determine how this unprecedented situation is affecting the professionals studied. For example, a lot of research is being done concerning other occupations such as health workers, teachers, etc. [5,55,56,57,58,59,60,61,62,63,64,65].

Many sociodemographic variables, such as subjective perceptions, increase the possibilities of suffering burnout in the security forces including its different subscales. We highlight the finding of (1) the need for psychological and psychiatric treatment; (2) the subjective perception of needing such treatment because of a new outbreak coronavirus, a situation that is currently being faced; (4) the need to establish psychological or psychiatric support services from work centers; (5) the absence of PPE; (6) anxiety in the face of death; and (7) the lack of recognition by the organizations for which they work.

The professionals reporting high levels of anxiety in the face of death, both in the set of subscales and for burnout, were found to be up to 2.2 times more likely to suffer from burnout. This shows the relationship between mortality, anxiety, and burnout. During the first wave of the pandemic in Spain, security and law enforcement professionals had to perform tasks that required contact with infected and dead people, as they were forced to disinfect centers, build field hospitals, transfer patients or bodies, provide health care, or have contact with infected and dead colleagues. They have also been direct witnesses to the enormous damage caused by the SARS-CoV-2, which has produced horrific scenes. It is therefore necessary to seek preventive actions against this problem [66,67], especially since it has been shown that these professional groups are more psychologically affected than the general population [68].

It is also significant the apparently paradoxical fact that the professionals who have felt most recognized by their organization during the first wave of the pandemic show a greater predisposition to feel a lack of professional fulfillment. It is not strange. There have also been cases of decorated wartime veterans who later indicated this circumstance, sometimes in the context of post-traumatic stress [69,70,71,72,73]. It may be, precisely, a consequence of this pandemic that is modifying so many patterns in our society, and that shows the impact it is having on these professionals. This is a topic that must continue to be analyzed in the future and for which more research is needed.

## 5. Conclusions

The novel processes and preventive measures that have been implemented to mitigate, as much as possible, the effects of the coronavirus have been named the new norm. In the groups studied here, measures must be taken to ensure that the high levels of stress experienced during the first wave of the pandemic do not also become the new normal. Working in situations of extreme stress should not be considered acceptable. This could cause the work performed by these professionals, in the context of the COVID-19 pandemic, to result in occupational disease. So, paradoxically, while these professionals protect the population by ensuring their safety and well-being, they may become ill in the eyes of the public institutions for which they work, with none of them taking decisive action for their protection.

Our findings highlight the relevance of conducting longitudinal research to report the existence of burnout syndrome over time, of implementing programs to reduce stress and promote healthy living habits, and of establishing preventive programs to identify professionals who present burnout and the phase in which they are within the police and military professionals.

Finally, this research has several limitations, including the difficulty of access, since it was only possible to approach these bodies through associations and unions in Spain. This situation may explain the lack of studies conducted with this group during the health crisis caused by COVID-19. However, it was necessary to establish a map of the current situation of the FFAA, FFCCS, and FFCCSE, whose role has been, along with the group of health workers, essential as they experienced a greater exposure to the virus. Another limitation found was that the proportion of women participating in the study was significantly less than that of men to establish a reliable gender comparison. Although this is a difficult issue to address because of the traditional disproportion of the percentage that exists in the professional group studied.

In short, the impact of the COVID-19 pandemic is being very important in the professional work of the group studied. Further studies will be necessary to determine its evolution along the successive waves. Only in this way will it be possible to obtain the most useful information to establish the best actions for the prevention of psychological problems in scenarios such as the one described. Which must always be based, of course, on an improvement in working conditions and the provision of sufficient material and human resources.

## Figures and Tables

**Table 1:** Description of research participants.

Variable	%
**Sex**	
Woman	12.7
Man	87.3
**Age (years)**	
Up to 30	18.4
31–40	34.6
41–50	34.6
51–60	11.5
>60	0.90
**Corp**	
Armed Forces	17.1
National Police (*Policía Nacional*)	41.5
Civil Guard (*Guardia Civil*)	36.7
Local Police	3.90
Other	0.80
*n* = 2182	

**Table 2:** Variables used in binary logistic regression.

**1. Sex**
Ref. Woman
(1) Man
**2. Age (Continuous, Years)**
Ref. Up to 30
31–40
41–50
51–60
>60
**3. Corp**
Ref. Other
Armed Forces
National Police
Civil Guard
Local Police
**4. Death anxiety (DA)**
Ref. No
Yes
**5. Need Psychological/Psychiatric Support (NPPS)**
Ref. No
Yes
**6. Psychological/Psychiatric Support Should be Offered from Workplaces (PPSS)**
Ref. No
Yes
**7. Psychological/Psychiatric Support may be Needed (PPSN)**
Ref. No
Yes
**8. Lack of PPE Increased your Stress and Anxiety Level (PPE)**
Ref. No
Yes
**9. Worked During the First Wave of the Pandemic (WFW)**
Ref. No
Yes
**10. Feel that his Work was Recognized (FWR)**
Ref. No
Yes

**Table 3:** Descriptive results. MBI, Maslach Burnout Inventory; PPE, personal protective equipment.

Emotional Exhaustion Subscale	%
Low	29.9
Medium	16.3
High	53.8
**Depersonalization Subscale**	
Low	17.4
Medium	24.5
High	58.0
**Personal Accomplishment Subscale**	
Low	46.3
Medium	26.6
High	27.1
**Total MBI**	
Yes	28.5
No	71.5
**Death Anxiety**	
Low	30.9
High	69.1
**Need Psychological/Psychiatric Support**	
Yes	26.2
No	73.5
**Psychological/Psychiatric Support Should be Offered in Workplace**	
Yes	88.2
No	11.8
**Psychological/Psychiatric Support may be Needed**	
Yes	52.5
No	47.5
**Lack of PPE Increased Stress and Anxiety Level**	
Yes	87.4
No	12.6
**Worked During the First Wave of the Pandemic**	
Yes	73.9
No	26.1
**Feel that Work was Recognized**	
Yes	10.4
No	89.6

**Table 4:** Summary of binary logistic regression models.

	B	Sig.	Exp (B)	95% CI Exp (B)	
				Lower	Superior
**Emotional Exhaustion Subscale**					
Age: 41–50 years	0.352	0.018	1.422	1.062	1.904
Armed Forces	1.899	0.032	6.678	1.175	37.941
National Police (*Policía Nacional*)	2.235	0.011	9.342	1.660	52.589
Civil Guard (*Guardia Civil*)	2.037	0.021	7.670	1.362	43.213
Local Police	2.135	0.020	8.457	1.400	51.092
NPPS	0.496	0.002	1.642	1.207	2.234
PPSN	0.694	0.000	2.002	1.570	2.552
PPE	0.460	0.002	1.585	1.187	2.116
DA	1.015	0.000	2.759	2.238	3.402
FWR	−0.388	0.017	0.678	0.494	0.932
Constant	−2.846	0.002	0.058		
**Depersonalization Subscale**					
Sex: Man	0.556	0.001	1.744	1.269	2.398
PPSN	0.322	0.009	1.380	1.083	1.758
DA	0.797	0.000	2.220	1.740	2.832
FWR	−0.426	0.015	0.653	0.463	0.922
Constant	−0.333	0.654	0.717		
**Personal Accomplishment Subscale**					
Sex: Man	−0.318	0.023	0.727	0.553	0.957
Age: 31–40 years	−0.388	0.004	0.679	0.523	0.881
NPPS	−0.519	0.000	0.595	0.470	0.753
PPSS	0.466	0.002	1.593	1.190	2.132
PPSN	−0.331	0.003	0.718	0.577	0.894
PPE	−0.492	0.001	0.611	0.454	0.823
DA	−0.504	0.000	0.604	0.495	0.738
FWR	0.265	0.018	1.304	1.046	1.624
Constant	2.220	0.010	9.210		
**MBI TOTAL**					
Age: 31-40	0.473	0.002	1.606	1.194	2.159
NPPS	0.636	0.000	1.888	1.479	2.409
PPSS	−0.389	0.020	0.678	0.489	0.940
PPSN	0.489	0.000	1.630	1.277	2.082
DA	0.805	0.000	2.236	1.762	2.837
Constant	−1.915	0.000	0.147		

## References

[1] Ministerio de Defensa Fuerzas Armadas Españolas. https://cutt.ly/OgOpsPI.

[2] Boletín Oficial del Estado Ley Orgánica 2/1986, de 13 de Marzo, de Fuerzas y Cuerpos de Seguridad. https://cutt.ly/pgOpfxw.

[3] López D.J., Del Río F.J., Ruíz A.M. (2014). Análisis psicométrico de la Escala de Soledad de UCLA (Versión 3) en una muestra de guardias civiles. Apunt. Psicol..

[4] Sheard I., Burnett M., St Clair-Thompson H. (2019). Psychological distress constructs in police with different roles. Int. J. Emerg. Serv..

[5] Martínez-López J.Á., Lázaro-Pérez C., Gómez-Galán J., Fernández-Martínez M.M. (2020). Psychological impact of COVID-19 emergency on health professionals: Burnout incidence at the most critical period in Spain. J. Clin. Med..

[6] Gribble R., Connelly V., Fear N.T. (2020). Living a life less ordinary: What UK military families can teach the families of essential workers responding to COVID-19. J. Mil. Veteran Fam. Health.

[7] Greenberg N., Docherty M., Gnanapragasam S., Wessely S. (2020). Managing mental health challenges faced by healthcare workers during COVID-19 pandemic. BMJ.

[8] Maslach C., Jackson S.E. (1981). MBI: Maslach Burnout Inventory Manual.

[9] Violanti J.M., Vena J.E., Petralia S. (1998). Mortality of a police cohort: 1950-1990. Am. J. Ind. Med..

[10] Martelli T.A., Waters L.K., Martelli J. (1989). The police stress survay: Reliability and relation to job satisfaction and organizational commitment. Psychol. Rep..

[11] García T. (2015). El estrés policial. Segur. Y Salud En El Trab..

[12] Brown J.M., Campbell E.A. (1994). Stress and Policing: Sources and Strategies.

[13] Rodríguez F., Arce R. (2016). Militares desplegados en misiones internacionales:percepción del estrés y síntomas asociados. Sanid. Mil..

[14] Gaibor J.E., Liger T.D., Safla J.P., Fernández M.I., Cholota L.P. (2018). El estrés en las Fuerzas Armadas: La situación de estrés en los aspirantes a soldados en la ESFORSE, promoción 2015–2017. Rev. De Cienc. De Segur. Y Def..

[15] Vojvodic A.R., Dedic G. (2018). Correlation between Burnout Syndrome and anxiety in military personnel. Serb. J. Exp. Clin. Res..

[16] Stevelink S.A., Jones N., Jones M., Dyball D., Khera C.K., Pernet D., MacCrimmon S., Murphy D., Hull L., Greenberg N. (2019). Do serving and ex-serving personnel of the UK armed forces seek help for perceived stress, emotional or mental health problems?. Eur. J. Psychotraumatol..

[17] Bartone P.T., Bowles S.V. (2020). Coping with recruiter stress: Hardiness, performance and well-being in US Army recruiters. Mil. Psychol..

[18] Lázaro-Pérez C., Martínez-López J.A., Gómez-Galán J. (2020). COVID-19 pandemic and death anxiety in security forces in Spain. Int. J. Environ. Res. Public Health.

[19] Stogner J., Miller B., McLean K. (2020). Police stress, mental health, and resiliency. Am. J. Crim. Justice.

[20] Caycho T., Carbajal C., Vilca L.W., Heredia J., Gallegos M. (2020). COVID-19 y salud mental en policías peruanos: Resultados preliminares. Acta Med. Peru..

[21] Frenkel M.O., Giessing L., Egger-Lampl S., Hutter V., Oudejans R., Kleygrewe L., Hutter V., Oudejans R., Plessner H. (2020). The impact of the COVID-19 pandemic on European police officers: Stress, demands and coping resources. J. Crim. Justice.

[22] (2020). Ministerio de Sanidad, Procedimiento de Actuación Para los Servicios de Prevención de Riesgos Laborales Frente a la Exposición al Nuevo Coronavirus (SARS-COV-2). https://cutt.ly/9gOi4NF.

[23] Asociación Unificada de Guardias Civiles La Guardia Civil Dobla en Número de Agentes Infectados Por el COVID-19 a los de la Policía Nacional. https://cutt.ly/jgOpgAc.

[24] Fuerzas y Cuerpos de Seguridad del Estado Coronavirus: Fuerzas y Cuerpos de Seguridad Piden que se les Clasifique de Alto Riesgo. https://cutt.ly/kgOyOYq.

[25] Barro P. Marlaska da Esquinazo a las Medallas Para los 21 Policías y Guardias Civiles Muertos por Covid. https://cutt.ly/VgOyD8N.

[26] Warsini S., West C., Ed G.D., Res G.C., Mills J., Usher K. (2014). The psychosocial impact of natural disasters among adult survivors: An integrative review. Issues Ment. Health Nurs..

[27] Fernández-Prada M., González-Cabrera J., Iribar-Ibabe C., Peinado J.M. (2017). Riesgos psicosociales y estrés como predictores del burnout en médicos internos residentes en el Servicio de Urgencias. Gac. Médica De México.

[28] Généreux M., Schluter P.J., Takahashi S., Usami S., Mashino S., Kayano R., Kim Y. (2019). Psychosocial management before, during, and after emergencies and disasters: Results from the Kobe expert meeting. Int. J. Environ. Res. Public Health.

[29] Moser D.A., Glaus J., Frangou S., Schechter D.S. (2020). Years of life lost due to the psychosocial consequences of COVID19 mitigation strategies based on Swiss data. Eur. Psychiatry.

[30] Gray B., Hanna F., Reifels L. (2020). The integration of mental health and psychosocial support and disaster risk reduction: A mapping and review. Int. J. Environ. Res. Public Health.

[31] García J.M., Herrero S., León J.L. (2007). Validez factorial del Maslach Burnout Inventory (MBI) en una muestra de trabajadores del Hospital Psiquiátrico Penitenciario de Sevilla. Apunt. De Psicol..

[32] Collett L., Lester D. (1969). The fear of death end the fear of dying. J. Psychol..

[33] Campedelli G.M., Aziani A., Favarin S. (2020). Exploring the Effect of 2019-nCoV Containment Policies on Crime: The Case of los Angeles. arXiv.

[34] Perez-Vincent S.M., Carreras E., Gibbons M.A., Murphy T.E., Rossi M.A. (2020). COVID-19 Lockdowns and Domestic Violence.

[35] Viglione J., Alward L.M., Lockwood A., Bryson S. (2020). Adaptations to COVID-19 in community corrections agencies across the United States. Vict. Offenders.

[36] Stickle B., Felson M. (2020). Crime rates in a pandemic: The largest criminological experiment in history. Am. J. Crim. Justice.

[37] Testoni I., Nencioni I., Ronconi L., Alemanno F., Zamperini A. (2020). Burnout, reasons for living and dehumanisation among Italian penitentiary police officers. Int. J. Environ. Res. Public Health.

[38] Ministerio del Interior La Policía Nacional actualiza el Plan de Actuación frente al COVID-19. https://cutt.ly/MgOi9zk.

[39] Mohler G., Bertozzi A.L., Carter J., Short M.B., Sledge D., Tita G.E., Uchida G., Brantingham P.J. (2020). Impact of social distancing during COVID-19 pandemic on crime in Los Angeles and Indianapolis. J. Crim. Justice.

[40] Prati G., Boldrin S. (2011). Fattori di stress e benessere organizzativo negli operatori di polizia penitenziaria. G. Ital. Med. Lavoro Ergon..

[41] Baudino M. (2014). La polizia penitenziaria tra sovraffollamento carcerario e burnout: Il dibattito interno. Rivista Criminol. Vittimol. Sicur..

[42] Linares O.L., Maldonado C.F., Gutiérrez R.E. (2018). Impacto de la satisfacción laboral en el desempeño de un grupo de policías estatales. Rev. Interam. De Psicol. Ocup..

[43] Violanti J.M. (1985). The police stress process. J. Police Sci. Adm..

[44] Malach-Pines A., Keinan G. (2006). Stress and burnout in Israeli border police. Int. J. Stress Manag..

[45] Wang Z., Inslicht S., Metzler T.J., Henn-Haase C., McCaslin S.E., Tong H., Neylan T.C., Marmar C.R. (2010). A prospective study of predictors of depressive symptoms in police. Psychiatry Res..

[46] Chambel M.J., Oliveira-Cruz F. (2010). Breach of psychological contract and the development of burnout and engagement: A longitudinal study among soldiers on a peacekeeping mission. Mil. Psychol..

[47] Vuorensyrjä M., Mälkiä M. (2011). Nonlinearity of the effects of police stressors on police officer burnout. Policing.

[48] Castro Y., Orjuela M., Lozano C., Avendaño B., Vargas N. (2012). Estado de salud de una muestra de policías y su relación con variables policiales. Divers. Perspect. En Psicol..

[49] Stearns S., Shoji K., Benight C.C. (2018). Burnout among US Military behavioral health providers. J. Nerv. Ment. Dis..

[50] Ruiz A.G. (2020). Burnout o síndrome del desgaste profesional en la Armada. Rev. Gen. De Mar..

[51] Nock M.K., Deming C.A., Fullerton C.S., Gilman S.E., Goldenberg M., Kessler R.C., McCarroll J.E., McLaughlin K.A., Peterson C., Schoenbaum M. (2013). Suicide among soldiers: A review of psychosocial risk and protective factors. Psychiatry Interpers. Biol. Process..

[52] Gupta S., Kohli K., Padmakumari P., Dixit P.K., Prasad A.S., Chakravarthy B.S., Shukla R., Ghana P., Mahapatra D., Varadaraj G. (2020). Psychological health among armed forces doctors during COVID-19 pandemic in India. Indian J. Psychol. Med..

[53] Gómez Galán J. (2020). The Black Death and other historical pandemics. Front. Sci..

[54] Ashton J. (2020). COVID-19 and the Spanish flu. J. R. Soc. Med..

[55] Ruiz-Fernández M.D., Ramos J.D., Ibáñez O., Cabrera J., Carmona M.I., Ortega A.M. (2020). Compassion fatigue, burnout, compassion satisfaction and perceived stress in healthcare professionals during the COVID-19 health crisis in Spain. J. Clin. Nurs..

[56] Joshi G., Sharma G. (2020). Burnout: A risk factor amongst mental health professionals during COVID-19. Asian J. Psychiatry.

[57] Dinibutun S.R. (2020). Factors associated with burnout among physicians: An evaluation during a period of COVID-19 pandemic. J. Healthc. Leadersh..

[58] Trumello C., Bramanti S.M., Ballarotto G., Candelori C., Cerniglia L., Cimino S., Crudele M., Lombardi L., Pignataro S., Viceconti M.L. (2020). Psychological adjustment of healthcare workers in italy during the COVID-19 pandemic: Differences in stress, anxiety, depression, burnout, secondary trauma, and compassion satisfaction between Frontline and Non-Frontline Professionals. Int. J. Environ. Res. Public Health.

[59] Matsuo T., Kobayashi D., Taki F., Sakamoto F., Uehara Y., Mori N., Fukui T. (2020). Prevalence of health care worker burnout during the coronavirus disease 2019 (COVID-19) pandemic in Japan. Jama Netw. Open.

[60] Luceño L., Talavera B., García-Albuerne Y., Martín J. (2020). Symptoms of posttraumatic stress, anxiety, depression, levels of resilience and burnout in Spanish health personnel during the COVID-19 Pandemic. Int. J. Environ. Res. Public Health.

[61] Khasne R.W., Dhakulkar B.S., Mahajan H.C., Kulkarni A.P. (2020). Burnout among healthcare workers during COVID-19 pandemic in India: Results of a questionnaire-based survey. Indian J. Crit. Care Med..

[62] Panisoara I.O., Lazar I., Panisoara G., Chirca R., Ursu A.S. (2020). Motivation and continuance intention towards online instruction among teachers during the COVID-19 pandemic: The mediating effect of burnout and technostress. Int. J. Environ. Res. Public Health.

[63] Mejía J.C., Silva C.A., Rueda Y.M. (2020). Ruta de atención psicosocial para docentes con síndrome de burnout a causa de la cuarentena generada por el COVID-19. Rev. De Investig. En Gestión Ind. Ambient. Segur. Y Salud En El Trab..

[64] Sokal L., Trudel L.E., Babb J. (2020). Canadian teachers’ attitudes toward change, efficacy, and burnout during the COVID-19 pandemic. Int. J. Educ. Res. Open.

[65] Shlenskaya N., Karnaukhova A., Son L., Lapteva E., Chen K.C. (2020). Teachers’ burnout in online university courses in the time of pandemic. The 4th International Conference on Education and Multimedia Technology.

[66] McCraty R., Atkinson M. (2012). Resilience training program reduces physiological and psychological stress in police officers. Glob. Adv. Health Med..

[67] Vojvodić A.R., Dedić G., Dukić-Dejanović S. (2019). Defense mechanisms and quality of life in military personnel with burnout syndrome. Vojnosanit. Pregl..

[68] Hartley T.A., Burchfiel C.M., Fekedulegn D., Andrew M.E., Violanti J.M. (2011). Health disparities in police officers: Comparisons to the U.S. general population. Int. J. Emerg. Ment. Health.

[69] Haas A.P., Hendin H. (1985). Wounds of War: The Psychological aftermath of Combat in Vietnam.

[70] Deahl M.P., Gillham A.B., Thomas J., Searle M.M., Srinivasan M. (1994). Psychological sequelae following the Gulf War. Factors associated with subsequent morbidity and the effectiveness of psychological debriefing. Br. J. Psychiatry.

[71] Rudd M.D., Goulding J., Bryan C.J. (2011). Student veterans: A national survey exploring psychological symptoms and suicide risk. Prof. Psychol. Res. Pract..

[72] Elliott T.R., Hsiao Y.Y., Kimbrel N.A., Meyer E.C., DeBeer B.B., Gulliver S.B., Kwok O.I., Morissette S.B. (2015). Resilience, traumatic brain injury, depression, and posttraumatic stress among Iraq/Afghanistan war veterans. Rehabil. Psychol..

[73] Vinokur A.D., Pierce P.F., Lewandowski-Romps L., Hobfoll S.E., Galea S. (2011). Effects of war exposure on air force personnel’s mental health, job burnout and other organizational related outcomes. J. Occup. Health Psychol..

